# Relationship between body mass index and family functioning, family communication, family type and parenting style among African migrant parents and children in Victoria, Australia: a parent-child dyad study

**DOI:** 10.1186/s12889-016-3394-1

**Published:** 2016-08-03

**Authors:** S. Cyril, J. Halliday, J. Green, A.M.N. Renzaho

**Affiliations:** 1 Centre for Cardiovascular Research and Education, School of Public Health and Preventive Medicine, Monash University, Level 5, Alfred Centre, 99 Commercial Road, Prahran, VIC 3004 Australia; 2 School of Social Sciences and Psychology, Western Sydney University, Penrith, 2751 NSW Australia; 3 School of Psychology, Deakin University, Burwood, Australia; 4 Murdoch Children’s Research Institute and Department of Paediatrics, The University of Melbourne and Parenting Research Centre, 323 Victoria Pde, East Melbourne, 3002 VIC Australia; 5 Humanitarian and Development Studies, School of Social Sciences and Psychology, Western Sydney University, Locked Bag 1797, Penrith, 2751 NSW Australia

**Keywords:** Family functioning, Intergenerational, Parenting, African migrants, Obesity, Children, Family communication, Family dynamics, Family type

## Abstract

**Background:**

Although childhood obesity prevalence is stabilised in developed countries including Australia, it is continuing to rise among migrants and socially disadvantaged groups in these countries. African migrants and refugees in particular, are at high risk of obesity due to changes in their family dynamics. The aim of this study was to examine the difference between children and parental perception of family functioning, family communication, family type and parenting styles and their relationship with body mass index.

**Methods:**

A cross-sectional parent-child dyad study was conducted among 284 African families from migrant and refugee backgrounds living in metropolitan Melbourne, Australia. Bilingual workers were trained to collect demographic, anthropometric and questionnaire data on family functioning, parenting, family type and family communication.

**Results:**

Parents and children reported different levels of family dynamics. Children reported a higher prevalence of poor family functioning (61.5 %, 95 % CI: 55.6, 67.2 versus 56.8 %, 95 % CI: 49.7, 61.6) and protective family type (29 %, 95 % CI: 23.9, 34.5 vs. 13.4 %, 95 % CI: 9.9, 17.9), but a lower prevalence of authoritative parenting style (51.6 %, 95 % CI: 45.7, 57.5 vs. 63 %, 95 % CI: 57.5, 68.8) than parents. There was a positive relationship between poor family functioning and child BMI both before (β = 1.28; 95 % CI: 0.14, 2.41; *p* < 0.05) and after (β = 1.73; 95 % CI: 0.53, 2.94; *p* < 0.001) controlling for confounders, and an inverse relationship between consensual family type and child BMI after adjustment (β = −1.92; 95 % CI: −3.59, −0.24; *p* < 0.05). There was no significant relationship between parental BMI and family functioning, communication, family type or parenting style.

**Conclusion:**

Children’s perception of poor family functioning was associated with childhood obesity. Family interventions to reduce childhood obesity need to adopt an intergenerational approach to promote a clear understanding of family dynamics between children and parents. Unless these intergenerational challenges associated with family dynamics are clearly addressed in obesity interventions, current obesity prevention initiatives will continue to widen the childhood obesity gap in Australia.

## Background

Current evidence shows that although childhood obesity rates are plateauing in developed countries, they are still rising among migrants and socially disadvantaged groups [1, 2]. Migrants originating from low and middle-income countries are at high risk of obesity when settling in developed countries [3]. African refugees and migrants are one population group who are at increased risk of obesity and obesity-related diseases [3, 4]. For example, African migrant children experience rapid catch-up growth and weight gain during the first 6 to 10 years of resettlement [5]. Migrants are also at higher risk of obesity compared to the host population in developed countries [6, 7]. Existing evidence suggests that acculturation – the cultural and psychological changes that occur when two cultures come into contact with each other [8, 9] increases the risk of obesity among migrants [3]. The effect of dietary acculturation among children is often exacerbated by pre-migration cultural beliefs and attitudes towards food, body size perception and eating behaviours which are strongly linked to unhealthy weight gain post-migration [10].

Acculturation not only affects the diet but also affects family dynamics and parent-children relations. Family dynamics refer to the pattern of interactions and relationships between family members [11, 12]. It involves various aspects such as family functioning, family communication patterns and parenting styles. Family functioning involves the emotional, physical and psychological activities between family members [13]. Poor family functioning has been found to be strongly associated with childhood obesity especially among migrants [14]. Family communication on the other hand is characterised by communication practices followed in families with poor communication leading to higher levels of obesity in children [15, 16]. Evidence shows that post-migration changes in family structure, dynamics and roles among African migrants re-settling in western countries such as Finland, Canada, and New Zealand have been reported [17–19].

Following migration, the changing relations and roles between husband and wife heightens intergenerational conflicts between adult migrants and their children. Fathers often experience internal conflict and difficulties regarding their roles as parents within the Australian society, and in shaping their relationships with their children [20]. On the other hand, children acculturate at a higher rate compared to parents, which causes parent-child conflicts. Renzaho et al [21] have found that the intergenerational acculturation gap contributes to poor relationships between parents and their children, ineffective feeding practices and tensions within the family. African migrants belong to the collectivist culture whose approach to parenting involves the older generation being in authority, with support from the immediate and extended family crucial to raising a child and resolving family conflicts [22]. Current evidence shows that migrant populations usually do not adapt their parenting styles as they integrate within the host society [23]. With parents being primary caregivers of children, it has been hypothesised that parenting styles may increase the risk of childhood obesity [24, 25]. Based on two dimensions of parental behaviour namely, control and warmth, four parenting styles - permissive, authoritarian, authoritative and neglectful have been described [26–29] (Table 1). Authoritative parents are those who are warm, exercise moderate levels of control and use inductive reasoning as a means of controlling children’s behaviour. Authoritarian parents employ high levels of control and no warmth. Permissive parenting describes those who are warm but exercise little control or supervision over their children. Parents who are neither warm nor exercise control over their children are termed unengaged or neglectful. African parents tend to remain authoritarian in their role [20]. Rhee et al [24] state that compared to children of authoritative parents, children of authoritarian parents have almost five times the odds and children of permissive and neglectful parents have twice the odds of being overweight. Current evidence also shows that among African migrants, lack of parental supervision and discipline is related to adolescent BMI [30].

**Table 1:** Parenting styles [67]

	High level of sensitivity and emotional involvement	Low level of sensitivity and emotional involvement
High demands for maturity and self-control	Authoritative parenting style (respectful of child’s opinions but sets limits)	Authoritarian parenting style (strict disciplinarian)
Low demands for maturity and self-control	Permissive parenting style (no discipline)	Neglectful parenting style (uninvolved)

The family communication patterns theory stipulates that family members reach an agreement by either conforming to other family members (*conformity orientation*) or by discussion and debate (*conversation orientation*) [31]. According to this theory, four family types have been identified - consensual, pluralistic, protective, and laissez-faire. In consensual families, there is a balance between open communication and agreement with the existing family hierarchy; in protective families obedience to family norms exists; in pluralistic families there is open unrestrained communication, whereas in laissez-faire there is minimal conversation and engagement by the family as a unit. The communication pattern in a family is determined by examining the extent to which the family relies on conversation orientation and conformity orientation (Table 2).

**Table 2:** Family communication patterns [31]

	High conversation orientation	Low conversation orientation
High conformity orientation	Consensual (balanced)	Protective (strict disciplinarian)
Low conformity orientation	Pluralistic (no discipline)	Laissez-Faire (uninvolved)

Family functioning affects many aspects of family life including: acceptance of individuals, consensus on decisions, communication, and the ability to solve day-to-day problems [13]. Post-migration, African women are sometimes required to seek employment to meet the financial needs of the family [20]. This challenges the traditional role of the man being the head of the family and decision maker. Men struggle to cope with domestic chores and often face an identity crisis. Families begin to see role reversals from the father as a disciplinarian and the mother as a nurturer [4, 20]. With changes to the family roles and relations, and absence of extended family in the host country, family functioning is affected [4, 13]. Family functioning affects childhood obesity by being an important factor in the regulation of children’s eating behaviours [32]. Poor family functioning is associated with an increased risk of obesity in children, and childhood obesity in turn impacts on the functionality of these families [15, 28, 29].

As described above, the current evidence suggests an association between family functioning, family communication, family type and parenting style, and childhood obesity among migrant populations. However, it is possible that parents and children are affected differently due to the fact that they perceive family functioning, family communication, family type and parenting style differently. More importantly, the current evidence linking family functioning, family communication, family type and parenting to obesity stems from parental data. To date, studies examining child data on this issue are lacking.

Therefore, the aim of this study was to examine the difference between children's and parental perception of family functioning, family communication, family type and parenting styles and their relationship with body mass index. We hypothesised that, there would be parental and children differences in the perception of family functioning, family communication, family type and parenting styles. We further hypothesised that there would be a difference in the relationship between family functioning, family communication, family type and parenting styles with BMI between African migrant parents and their offspring.

## Methods

### Study design, sample and recruitment

Data were obtained from 284 African parents and their children aged between 12 and 17 years, living in metropolitan Melbourne, Australia. Participants were from both migrant and refugee backgrounds and were recruited using two complementary strategies. The first strategy involved media advertising: advertisements in local newspapers, radio announcements in the migrant communities’ first language, and flyers and posters in African languages displayed and handed out at community associations, health centres and restaurants. The media advertisements included a ‘call’ to participate, contact details for the study, and images of Melbourne-based community leaders who endorsed the study. This strategy was unsuccessful in engaging African families to participate. The second strategy involved recruiting and training bilingual community workers, who in turn mobilised African migrant communities for the study. Bilingual workers were engaged from a range of African communities across the suburbs of Melbourne to ensure diversity in the sample. The bilingual workers mobilized eligible families from their networks using snowball sampling techniques. African families were more eager to take part in the study when bilingual workers were from their own community; this approach was far more successful than media advertisements.

### Data collection procedure and study governance

Only households with at least one child aged between 12 and 17 years were included in the study. Where a household had more than one eligible child, the participating child was randomly selected, and both the child and their primary carers were interviewed. Before the interview could commence, each responding carer provided written consent for themselves and their eligible child. Data were collected by trained bilingual workers. They participated in the training run by the project team prior to undertaking participant recruitment and data collection. The training included ethical and safety considerations (e.g. privacy and confidentiality, informed consent), interview techniques, the accurate measurement of height and weight using the study equipment, and familiarisation with the study materials. The training was complemented with practice interviews to ensure adherence to interview protocols and to help interviewers refine their interviewing technique. The study was overseen by the African Review Panel, a community advisory committee comprising African community leaders, lay representatives and health professionals working with African communities [33]. This culturally appropriate method has been successfully used in our previous studies [20, 32].

### Study variables

#### Dependent variables

The dependent variable was the body mass index obtained from height and weight measurements. Height (to the nearest 0.01 cm) and weight (to the nearest 0.1 kg), without shoes, were measured for each participant using a portable stadiometer (Wedderburn WS-HRP Portable Height Rods) and physician’s scale (Tanita HD-327, Japan). Body mass index (BMI) was calculated using the formula: weight (kg) divided by height (m) squared. For parents, BMI data were used to classify underweight (<18.50 kg/m^2^), normal range (18.50–24.99 kg/m^2^), overweight (≥25.00–29.99 kg/m^2^) and obesity (≥30.00 kg/m^2^) as recommended by WHO [34]. For children, BMI-for-age Z -scores (BMIz) were calculated using the 2006 WHO growth references [35]. The prevalence of thinness, overweight and obesity was computed using recommended WHO cut-off points based on the BMIz [36].

#### Independent variables

##### Family functioning

Family functioning was measured among both parents and children using the general family functioning subscale of the McMaster Family Assessment Device (FAD) [37], which includes 12 items scored on a 4 point Likert scale (1 = strongly agree; 2 = agree; 3 = disagree; 4 = strongly disagree). The psychometric properties of the FAD have been established in various studies and found to have strong internal consistency in various settings (α 0.80 to 0.89) [13, 38, 39]. In this study, the Cronbach’s alpha of FAD among children was α = 0.82 and among parents was α = 0.78. After reverse scoring positive items, a mean score was calculated by summing the item scores and dividing the total by the number of items. In order to compute the prevalence of poorly functioning households, the mean family functioning scores were dichotomized into well-functioning (scores ≤2) and poorly functioning families (>2) [13]. Higher mean scores indicated poorer family functioning [37].

##### Family communication and family type

Family Communication was measured using the Revised Family Communication Pattern (RFCP) questionnaire which consists of 26 statements about communication behaviour and attitudes within the family, with two slightly different versions existing for parents and children. The RFCP is a valid and reliable measure of family communication attitudes and behaviour with child-parent pairs. Cronbach alpha on the conversation scale was 0.88 for children and 0.81 among parents, the conformity scale showed α = 0.72 among children and α = 0.74 among parents. Two dimensions of family communication patterns (socio-orientation and concept-orientation) were used to identify four family types namely, a) *pluralistic families* which stress the relationship between child and concepts who score high on concept-orientation but low on socio-orientation b) *protective families* which stress a relationship of obedience and conformity between child and parents who score low on concept-orientation but high on socio-orientation c) *consensual families* who strive for a balance between conformity and openness who score high on both scales and d) laissez-faire families with minimal communication between child and parents scoring low on both scales [40].

##### Parenting style

A single item developed by Radziszewska et al. [41] was used to measure parenting style among both parents and children, based on the authoritarian, authoritative, permissive, and unengaged types. The Cronbach alpha was 0.84 for children and 0.81 for parents in this study.

### Demographic and socioeconomic variables

Demographic and socioeconomic factors included age in years, gender (1 = boy; 0 = girl), length of stay in Australia in years, religion (0 = Christian, 1 = Muslim, and 2 = Other), education (0 = High school or less, 1 = Completed year 12, 2 = Tertiary education, 3 = Still attending school), and household income (0 = <$15,000, 1 = $15–25,000, 2 = $26–35,000, 3 = $36–45,000, and 4= >45,000)

### Data analysis

Data were analysed using the software Stata version 12 [42]. Frequencies between groups were compared by chi-square tests while group means were compared using t-tests (Table 3). In the first instance, linear regression models were used to assess the relationship between demographic and socioeconomic factors and BMI and to identify factors to adjust for when examining the relationship between family dynamics and BMI (Table 4). Multiple linear regression models were used to assess the relationship between BMI and family functioning, family communication, family type and parenting style; adjusting for confounding factors (Table 5). The significance was set at *p* < 0.05.

**Table 3:** Perceptions of family functioning, family communication, family type and parenting style among children and parents

	Children			Parents			*P*-values
	N	%	95 % CI	N	%	95 % CI	
Family functioning							χ2 = 1.865, *p* = 0.172
Well-functioning	105	38.5	32.8, 44.4	119	44.2	38.4, 50.3	
Poorly functioning	168	61.5	55.6, 67.2	150	56.8	49.7, 61.6	
Mean (SD)	273	2.05(0.49)	1.99, 2.10	269	1.97 (0.45)	1.92, 2.02	0.0612
Family communication							
Conformity orientation (Mean (SD))	284	31.51 (5.30)	30.9, 32.1	284	31.49 (5.69)	30.8, 32.2	0.970
Conversation orientation (Mean (SD))	284	37.56 (7.76)	36.7, 38.5	284	40.66 (7.05)	39.8, 41.5	<0.001
Family type							χ2 = 26.90, *p* < 0.001
Laissez-faire	66	23.2	18.7, 28.5	54	19.0	14.8, 24.0	
Pluralist	57	20.1	15.8, 25.2	81	28.5	23.5, 34.1	
Consensual	79	27.8	22.9, 33.4	111	39.1	33.5, 44.9	
Protective	82	28.9	23.9, 34.5	38	13.4	9.9, 17.9	
Parenting style							χ2 = 10.50, *p* < 0.05
Authoritarian	85	30.5	25.3, 36.2	74	26.6	21.7, 32.2	
Authoritative	144	51.6	45.7, 57.5	176	63.3	57.5, 68.8	
Permissive	42	15.1	11.3, 19.8	22	7.9	5.3, 11.8	
Unengaged	8	2.9	1.4, 5.7	6	2.2	1.0, 4.7

**Table 4:** Relationship between demographic and socioeconomic factors and child and parental BMI

	Child BMI						Parent BMI					
	Uβ	95 % CI		Aβ	95 % CI		Uβ	95 % CI		Aβ	95 % CI	
Parental BMI	0.11*	0.00	0.22	0.15*	0.01	0.28	-	-		*-*	-	-
Gender, Male	0.52	−0.60	1.64	0.69	−0.67	2.05	−1.39*	−2.67	−0.12	−1.07	−2.84	0.69
Age	0.42**	0.11	0.72	0.47*	0.10	0.85	0.14***	0.06	0.22	0.11*	0.01	0.21
Length of stay in Australia	−0.05	−0.17	0.07	−0.19*	−0.36	−0.03	0.29***	0.17	0.40	0.24*	0.04	0.44
Religion, Muslim^a^	0.42	−0.84	1.68	−0.10	−1.75	1.56	3.19	1.86	4.52	2.99***	1.26	4.72
Religion, Other^a^	2.50	−0.75	5.76	−1.47	−6.31	3.36	1.56	−1.87	5.00	0.39	−5.60	6.39
Parent education completed year 12^b^	−0.36	−2.22	1.51	−0.53	−2.88	1.83	1.11	−0.92	3.14	0.05	−2.59	2.68
Parent education, Tertiary education^b^	0.14	−1.04	1.32	−0.18	−1.76	1.41	−0.57	−1.85	0.72	−1.33	−3.09	0.4
Parent education, Still at school^b^	6.06	−3.00	15.12	9.11	−0.21	18.42	−12.02**	*−21.90*	*−2.14*	−7.96	−18.02	2.09
Household income, $15–24,000^c^	−0.46	−2.08	1.15	−0.38	−2.22	1.46	−0.23	−2.04	1.58	0.07	−1.99	2.13
Household income, $25–34,000^c^	0.20	−1.39	1.78	0.05	−2.13	2.24	0.34	−1.44	2.11	0.72	−1.70	3.14
Household income, $35–44,000^c^	0.27	−1.57	2.11	−0.41	−2.54	1.73	−0.67	−2.72	1.39	−0.73	−3.08	1.62
Household income, >45,000^c^	−0.24	−2.36	1.87	0.74	−2.01	3.49	−0.16	−2.49	2.16	−1.76	−4.77	1.25

^a^Reference = Christian; ^b^Parental education level, reference = some high school or less; ^c^Household annual income, reference = <$15,000. * < 0.05; ** < 0.01; *** < 0.001; *U*β Unadjusted β coefficient, *A*β Adjusted β coefficient (adjusted for factors in the table)

**Table 5:** Relationship between family functioning, family communication, family type & parenting style and adolescent/parental BMI

	Children						Parents					
	Uβ	95 % CI:		Aβ	95 % CI		Uβ	95 % CI		Aβ	95 % CI	
Poorly functioning family	1.28*	0.14	2.41	1.73***	0.53	2.94	0.43	−0.81	1.67	0.47	−0.89	1.84
Family communication: Conformity orientation	−0.11*	−0.22	0.00	−0.11	−0.24	0.01	−0.03	−0.14	0.08	0.02	−0.10	0.14
Family communication: Conversation orientation	−0.04	−0.12	0.03	−0.08*	−0.15	0.00	−0.03	−0.12	0.06	−0.02	−0.12	0.08
Family type: Pluralist	−1.26	−2.92	0.40	−1.10	−2.86	0.66	−0.44	−2.21	1.32	−0.28	−2.18	1.62
Family type: Consensual	−1.47	−3.00	0.06	−1.92*	−3.59	−0.24	−0.83	−2.49	0.83	0.01	−1.82	1.83
Family type: Protective	−1.38	−2.91	0.15	−0.66	−2.33	1.01	−0.46	−2.58	1.67	0.26	−2.08	2.59
Parenting style: Authoritative	0.05	−1.23	1.32	0.21	−1.16	1.58	−0.21	−1.61	1.19	0.21	−1.36	1.79
Parenting style: Permissive	0.47	−1.32	2.25	0.23	−1.75	2.21	0.46	−1.97	2.89	2.34	−0.32	5.01
Parenting style: Unengaged	1.38	−2.02	4.77	1.35	−2.48	5.17	−2.06	−6.67	2.56	0.72	−9.24	10.68

Variables in the adjusted model include age, gender, length of stay in Australia, religion, parental education, and household income. *U*β Unadjusted β coefficient, *A*β Adjusted β coefficient. *p* values * < 0.05; ** < 0.01; *** < 0.001

## Results

### Sample characteristics

Participants’ demographic characteristics are outlined in Table 6. Total sample size was 568 with an equal number of children and parents (284 parents and 284 children). Mean (standard deviation) ages of children and parents were 14.7 years (1.87) and 39.6 years (7.86), respectively. The average length of stay in Australia was 5.76 years (3.37) for children and 7.3 (4.91) years for parents. More than one-third of parents had completed tertiary education while almost 40 % earned an annual income of less than $15,000. The mean BMI (SD) was 21.74 (4.60) kg/m^2^ among children and 27.73 (4.98) kg/m^2^ among parents. The prevalence of overweight and obesity was respectively 24.6 % (95 % CI: 19.8, 30.1) and 9.8 % (95 % CI: 6.7, 13.9) among children and 45.6 % (95 % CI: 39.8, 51.5) and 24.4 % (95 % CI: 19.7, 29.8) among parents. A further 10.5 % (95 % CI: 7.4, 14.7) of children and 0.7 % (95 % CI: 0.2, 2.8) of parents were classified as underweight.

**Table 6:** Demographic characteristics in children and parents

	Children		Parents	
	N	Mean (SD)	N	Mean (SD)
Age; (years)	283	14.72 (1.87)	281	39.66 (7.86)
Length of stay; (years)	250	5.76 (3.37)	276	7.32 (4.91)
	N	%	N	%
All	284	100	284	100
Gender				
Female	170	60.1	195	68.7
Male	113	39.9	89	31.3
Religion				
Christian	209	73.9	202	71.9
Muslim	69	24.3	71	25.3
Other	5	1.8	8	2.9
Education				
High school or less			148	53.1
Completed year 12			29	10.4
Tertiary education			101	36.2
Still attending school	278	100.0	1	0.4
Household income				
< $15,000	102	39.5	102	39.5
$15–25,000	48	18.6	48	18.6
$26–35,000	50	19.4	50	19.4
$36–45,000	33	12.8	33	12.8
> 45,000	25	9.7	25	9.7
BMI		21.74 (4.6)		27.73 (4.98)
Underweight (<18.50 kg/m^2^)	29	10.5 (95 % CI: 7.4, 14.7)	2	0.7 (95 % CI: 0.2,2.8)
Normal (18.50–24.99 kg/m^2^)	152	55 (95 % CI: 49.1, 61)	82	29.3 (95 % CI: 24.6, 35.4)
Overweight (≥25–29.99 kg/m^2^)	68	24.6 (95 % CI: 19.8, 30.1)	124	45.6 (95 % CI: 39.8,51.5)
Obesity (≥30.00 kg/m^2^)	27	9.8 (95 % CI: 6.7,13.9)	68	24.4 (95 % CI: 19.7, 29.8)

### Parental and child perceptions of family functioning, family communication, family type and parenting style

There were differences in parental and child perceptions of family functioning and parenting, with 61.5 % (95 % CI: 55.6, 67.2) of children versus 56.8 % (95 % CI: 49.7, 61.6) of parents classifying their families as poorly functioning, and 51.6 % (95 % CI: 45.7, 57.5) of children versus 63 % (95 % CI: 57.5, 68.8) of parents reporting an authoritative style of parenting (Table 3). Thirty percent of children (95 % CI: 25.3, 36.2) and 26.6 % (95 % CI: 21.7, 32.2) of parents reported an authoritarian parenting style, while 15 % (95 % CI: 11.3, 19.8) of children and 7.9 % (95 % CI; 5.3, 11.8) of parents reported a permissive style of parenting. Chi square test revealed that there was a significant difference (*p* <0.05) between children’s and parents’ perception of parenting style. The mean (SD) for conversation orientation of family communication was 37.5(7.7) in children and 40.6 (7.05) in parents, while for conformity orientation it was 31.5(5.3) among children and 31.5 (5.7) among parents. The difference between children and parental perception on conversation orientation type of family communication was significant (*p* <0.001). More than a quarter (27.8 %, 95 % CI: 22.9, 33.4) of children and 39 % (95 % CI: 33.5, 44.9) of parents reported a consensual family type while 29 % (95 % CI: 23.9, 34.5) of children and 13.4 % (95 % CI: 9.9, 17.9) of parents reported a protective family type. There was a significant difference (*p* <0.001) between children and parental perception of family type.

### Relationship between socio-demographic factors and child and parent BMI

In the adjusted analysis, factors positively associated with childhood obesity were older age (β = 0.47; 95 % CI: 0.10, 0.85; *p* < 0.05), and parental BMI (β = 0.15; 95 % CI: 0.01, 0.28; *p* < 0.05), while length of stay in Australia (β = −0.19; 95 % CI: −0.36, −0.03; *p* < 0.05) was inversely associated with childhood obesity. Among parents, BMI was positively associated with age (β = 0.11; 95 % CI: 0.01, 0.21; *p* < 0.05), length of stay (β = 0.24; 95 % CI: 0.04, 0.44; *p* < 0.05) and being a Muslim (β = 2.99; 95 % CI: 1.26, 4.72; *p* < 0.001).

### Relationship between family functioning, family communication, family type, parenting style, and obesity

After adjusting for confounders such as demographic and socio-economic factors, child BMI was positively associated with poor family functioning (β = 1.73; 95 % CI: 0.53, 2.94; *p* < 0.001) but inversely associated with conversation orientation type of family communication (β = −0.08; 95 % CI: −0.15, 0.00; *p* < 0.05) and consensual family type (β = −1.92; 95 % CI: −3.59, −0.24; *p* < 0.05). However, these results were only significant in the multivariate analyses and not evident in the univariate analyses, suggesting that the relationship between child BMI and family communication patterns could be influenced by other factors (i.e. its effect is only significant in the context of the other independent variables). Family functioning, family communication, family type and parenting style were not associated with parent BMI.

## Discussion

The aim of the study was to examine the difference between children's and parental perception of family functioning, family communication, family type and parenting styles and their relationship with body mass index. The reported prevalence of overweight and obesity (24.6 and 9.8 % respectively) among African children was higher than that found among Australian children (18 and 7 % respectively) [43]. The prevalence of overweight among African parents (45 %) was also higher when compared to their Australian counterparts (35 %) however the obesity rate among African parents (24.6 %) was lower than that found in Australian adults (28 %) [44]. Given that, if left unattended, childhood obesity often persists into adulthood [45], interventions to address childhood obesity among African migrant children are urgently needed. We found that child BMI was significantly associated with family-related factors, namely family functioning and family type. These findings suggest that effective childhood obesity prevention programs among African migrants need to take into account pre-migration experiences and be family-based.

We hypothesised that, there would be differences between parental and children’s perception of family functioning, family communication, family type and parenting styles. The hypothesis was partially confirmed. We found significant differences in the perception of family communication, family type and parenting styles between parents and children. These findings could be closely related to the intergenerational acculturation gap, which is the differing rates at which migrant parents and their children acculturate [46, 47]. Evidence shows that the intergenerational acculturation gap contributes to poor relationships between parents and children and is a significant source of intergenerational and intra-familial conflicts [10]. The independence sought by older children and interdependence expected by their parents, have been postulated as main reasons for this conflict [46]. African families hail from a collectivist culture and studies have shown that immigrant children from collectivist cultures experience greater intergenerational conflict compared to their native counterparts [46]. Evidence shows that intergenerational differences among immigrant families can be substantial when families face economic hardships and unemployment, a feature common among post-migration families [48]. In this study, more than one-third of the families were from a low income group earning less than $15,000 per annum. Economic disadvantage in itself and in interaction with intergenerational conflicts can promote obesogenic environments [10, 49]. Majority of the children in our study reported a protective family type, while parents reported a consensual family type. Protective patterns are centered on obedience to rules and conformity whereas consensual pattern involves a balance between conformity and openness. Studies have shown that discrepancies in the perception of family environment and communication often exist between children and their parents [30, 50].

Smokowski et al [51] found that intergenerational conflicts are related to family functioning and predicted by lower family cohesion. Several reasons have been postulated for poor family functioning amongst migrant families. The cultural distance between the culture of origin and that of the new society as well as changing relationships and roles between the husband and his wife in the new society can threaten the harmony of immigrant family relations and thus contribute to family dysfunction [20, 46]. Post- migration, due to the influence of school and peers, African children come to desire a lean body shape which conflicts with their parents’ desire to achieve a culturally appropriate ‘heavy, big or powerful’ body image [10]. These attitudes could widen the intergenerational acculturation gap leading to intergenerational conflict and family dysfunction [52]. However, more research is needed in this area, since the evidence determining whether family dynamics lead to intergenerational conflicts or these conflicts cause problems in family functioning, is not conclusive [51, 53].

Traditionally, African parents adopt an authoritarian style of parenting. Authoritarian styles have been linked to a higher risk of obesity in children [54]. However, in this study, an authoritative parenting style was most commonly reported by both African parents and children. This is in contrast with the literature which reports that over time migrant parents are less likely to make a transition from the authoritarian parenting style to the authoritative parenting style [55]. Studies also show that migrants who do not acculturate often tend to use authoritarian parenting styles [56]. With the average length of stay being 6.5 years in Australia post-migration, it is possible that the African families in our study are at varying stages of acculturation which could have led the transition to an authoritative parenting style [54]. Although the authoritative parenting style has been associated with lower child BMI, studies have found that it accounts for 7.4 % of the variance in children’s BMI, indicating that children from families adopting authoritative parenting style are also at increased risk for obesity [15, 57]. Our study did not find any significant association between parenting style and child or parental BMI.

We further hypothesised that there would be a difference in the relationship between family functioning, family type and parenting styles with BMI between African migrant parents and their offspring. The hypothesis was partially confirmed. Only family type and family functioning were associated with childhood obesity. We found that poor family functioning was positively associated with increasing BMI among children. The existing literature shows that poor family functioning accounts for 14–24 % variance in child BMI, poor health behaviours and deleterious health outcomes among children [28, 30]. Further family functioning also influences the eating behaviours of children through parental dietary practices, choice of foods and family meal environments [58]. Unless interventions targeting childhood obesity reduction undertake family-based approaches, successful achievement of program outcomes are unlikely [59]. Parental data from our study showed that consensual family type was associated with lower risk of childhood obesity. Studies show that both protective and laissez-faire family types are linked with poor eating behaviours among children, while consensual and pluralist family types have an optimal engagement with their children regarding their nutritional environment and have a greater chance of establishing childhood obesity preventive behaviours [60, 61]. A recent study by Hancock et al [62] has found that maternal protectiveness is associated with an increased risk of childhood obesity. Studies have shown that protective and unengaged family communication patterns were associated with maladaptive eating behaviours in children [63, 64]. However, most of these studies used parental data and failed to obtain data from children. Despite most children in our study reporting a protective communication pattern, we did not find any significant associations between protective family communication and obesity.

## Conclusion

This study found that family functioning and family type were associated with obesity among African migrant children in Australia. Using both parental and child data has contributed to a greater understanding of the differences in the perceptions of family dynamics between parents and children, prevalent in this population, and their relationship with child BMI. Unless these differences are clearly addressed in childhood obesity interventions, it is likely that the current obesity prevention initiatives will fail to effectively address childhood obesity among African migrants, which in turn would allow widening of the obesity-related disparities in Australia.

By adopting an intergenerational approach which incorporates a clear understanding of family functioning, family communication, family type and parenting styles, policymakers will be better able to tailor obesity programs to reduce migration-related inequalities in obesity. Given that a bidirectional relationship between family functioning and childhood obesity exists, such targeted approaches will ultimately facilitate the achievement of health outcomes in relation to obesity prevention.

### Strengths and limitations

The majority of the current literature surrounding childhood obesity has relied primarily on data collection from parents owing to the difficulty in recruiting children and collecting data from them [65]. However, our study involved collecting data from parent-child dyads which allowed us to compare and contrast information from both parents and children from the same families. Evidence has found that there has been no consistency in the information collected from parents on their children’s obesity levels and the actual BMI of the child [66], thus stressing the need for collecting information related to childhood obesity from children themselves. The current study collected data on children’s perspectives instead of solely relying on parents’ opinions. An additional strength of this study is the use of bilingual workers which enhanced participant recruitment, ensured adequate data collection and minimised missing data.

Being a cross-sectional study, causality cannot be assumed. Environmental factors such as school teachers, peers, friends and broader community which although important in influencing childhood obesity and influence children’s views on parenting, were not examined in this study. Given the increased risk of both intergenerational conflicts and poor family functioning amongst migrants, longitudinal studies investigating the causal relationship between intergenerational conflicts and family functioning in this group, as well as their combined causative role in the risk of childhood obesity are urgently needed. Such studies will advance our understanding of the role family dynamics and intergenerational conflicts play in the pathogenesis of childhood obesity.
